# Supplementary material for: Machine Learning Models Predicting Cardiovascular and Renal Outcomes and Mortality in Patients with Hyperkalemia
Source: Nutrients. 2022 Nov 3;14(21):4614. doi: 10.3390/nu14214614 (PMC9658112; doi:10.3390/nu14214614)
Supplement: Supplementary file 1 [file nutrients-14-04614-s001.zip › nutrients-1918431-supplementary.pdf]

# APPENDIX

**APPENDIX** .......... **1**
**Method details** .......... **2**
Data sources .......... 2
Machine learning algorithms .......... 2
Feature importance, selection of clinical variables .......... 3
Shapley additive explanations .......... 3
Reporting checklist .......... 3
**Supplementary tables and figures** .......... **4**
**Table S1. Clinical variables used as predictors for the model** .......... 4
**Table S2. Definitions of primary and secondary clinical outcomes** .......... **8**
**Table S3. Definitions of exploratory clinical outcomes** .......... **9**
**Table S4. Tuning hyperparameters for each modeling algorithm** .......... **10**
(a) Hyperparameters for the extreme gradient boosting model .......... 10
(b) Hyperparameters for the logistic regression model .......... 11
(c) Hyperparameters for the neural network model .......... 11
**Table S5. Prediction performance of the machine learning models for exploratory outcomes on the internal validation set** .......... **12**
**Table S6. Prediction performance of the extreme gradient boosting models on the external validation set based on the restricted condition** .......... **13**
**Table S7. TRIPOD Checklist** .......... **14**
**Figure S1. Data handling of the internal dataset** .......... **16**
**Figure S2. Patient flow diagrams for external validation** .......... **17**
**Figure S3. Receiver operator characteristics curve for exploratory outcomes evaluated on the internal dataset** .......... 18
**Figure S4 Receiver operator characteristic curves of the machine learning model in the external validation set** .......... 20
**Figure S5. Sensitivity analysis in the subgroup of patients in the external validation set applying the same outcome definition used in the derivation set.** .......... **21**
(a) Patient flow diagram .......... 21
(b) Receiver operator characteristics curve .......... 21
(c) Kaplan-Meier plots of high- and low-risk groups based on risk predictions .......... 22
**Fig. S6. Kaplan-Meier plots of high- and low-risk groups based on risk predictions in the**

**external validation set based on the restricted condition.** ..........................................................**23**
**References**..................................................................................................................**24**

## Method details

### Data sources

The MDV database consists of health insurance claims from Diagnosis Procedure Combination (DPC) hospitals, with consent obtained from the hospitals. It is one of the largest datasets of hospital medical procedures, diagnoses, laboratory data and prescriptions available in Japan. The dataset now covers over 30 million patients treated at >370 hospitals across Japan, including both in- and out-patients.

For external testing of the machine learning model developed, we used the RWD database maintained by the Health, Clinic and Education Information Evaluation Institute (Kyoto, Japan) with support by Real World Data Co., Ltd. This database is comprised of electronical medical records collected from approximately 160 hospitals across Japan. This dataset includes information on patient demographics, hospital diagnoses, prescriptions, procedures, and examinations, as well as laboratory data, covering approximately 20 million patients from both in- and out-patient clinics. Both MDV and RWD databases use ICD-10 codes.

### Machine learning algorithms

LR is a classical linear regression model widely used in medical research. The advantages of LR are its simplicity for model interpretation and robustness; thus, we used it as the reference to compare the prediction performance of the three algorithms. Before feeding data into the LR model, we standardized the variables by scaling to unit variance, and imputed missing values with the mean for each variable. No interaction term was addressed in the model.

Compared to the LR model, XGB generates an ensemble model[1] consisting of several decision tree models and automatically imputes missing values internally. This approach is effective when there are several types of relationships between explanatory variables and objective variables dependent on other variables. We suspected relationships with some effect modifiers exist between hyperkalemia and clinical outcomes; thus, the ensemble models were expected to predict the outcomes better. Early stopping was introduced to suppress overfitting.

NN modeling is a classical method for predicting outcomes using complex non-linear models. Recently, their performance has been much improved by increasing the model layers. In this study, more than 90 potential variables, some of which assumed to have non-linear linear relationships with the clinical outcomes, were available to develop the model. Therefore, we adopted NN to represent these nonlinearities between explanatory variables and objective variables. We used a dense NN with hidden layers of sizes 1,000, 200, and 15 neurons. The hidden layers used batch

normalization and ReLU activation[2], and missing values were set to zero. The learning rate was tuned as part of the fitting process.

**Feature importance, selection of clinical variables**

To select the predictors used in the phase-two model, we first summarized the variable importance of all outcomes for each variable. Importance values for each clinical variable were converted to rank ascending order, then each variable's rank was summed among all outcomes. If the summed variable importance ranks were lower than 20%, these clinical variables were set as candidates for deletion. Some of the variables that were clinically similar to, or were combinations of, other variables were also set as candidates for deletion, even though their summed feature importance rank values were high. We experimentally built models by excluding the candidates for deletion in order to determine the final set of variables as far as the performance of the model was maintained based on assessment of the area under the receiver operator characteristics curves.

**Shapley additive explanations**

SHAP (SHapley Additive exPlanations)[3] is a method of explaining the output of machine learning model. The goal of SHAP is to explain the prediction of an instance by computing the contribution of each feature to the prediction. The SHAP explanation method computes Shapley values, which interpret the impact of having a certain value for a given feature in comparison to the prediction we would make if that feature took some baseline value. We used the library[4] to calculate SHAP values for our models. The algorithms of calculating SHAP values can be found in for XGB[5] and for LR.[3]

**Reporting checklist**

This article is written following the TRIPOD (Transparent Reporting of a Multivariable Prediction Model for Individual Prognosis or Diagnosis) guidelines,[6] which are further elaborated in Table S6.

## Supplementary tables and figures

### Table S1. Clinical variables used as predictors for the model

### (a) Demographics and laboratory tests with observed rates

| Feature | Definition | Observed | Rate |
|---|---|---|---|
| ***Demographics*** | | | |
| Age | Age at index date (at least 18). | 8,752 | 100.0% |
| Sex | Male or female. | 8,752 | 100.0% |
| ***Laboratory tests*** | | | |
| Serum potassium level | Serum potassium level at index date. | 8,752 | 100.0% |
| eGFR | The minimum eGFR on the last day with eGFR records between 360 days before the index date (inclusive) and the index date (inclusive), ignoring all eGFR values greater than or equal to 1,000, or 'missing' if there is no such record. | 8,750 | 99.98% |
| HbA1c | The maximum HbA1c on the last day with HbA1c records between 360 days before the index date (inclusive) and the index date (inclusive), or 'missing' if there is no such record. | 6,160 | 70.38% |
| Total cholesterol | The maximum total cholesterol on the last day with total cholesterol records between 360 days before the index date (inclusive) and the index date (inclusive), or 'missing' if there is no such record. | 5,571 | 63.65% |
| HDL cholesterol | The maximum HDL cholesterol on the last day with HDL cholesterol records between 360 days before the index date (inclusive) and the index date (inclusive), or 'missing' if there is no such record. | 6,031 | 68.91% |
| LDL cholesterol | The maximum LDL cholesterol on the last day with LDL cholesterol records between 360 days before the index date (inclusive) and the index date (inclusive), or 'missing' if there is no such record. | 5,508 | 62.93% |
| Triglyceride | The maximum triglyceride on the last day with triglyceride records between 360 days before the index date (inclusive) and the index date (inclusive), or 'missing' if there is no such record. | 6,497 | 74.23% |
| Brain natriuretic peptide | The maximum brain natriuretic peptide on the last day with brain natriuretic peptide records between 360 days before the index date (inclusive) and the index date (inclusive), or 'missing' if there is no such record. | 2,395 | 27.37% |

## (b-1) Diagnoses and medications with positive rates (1/3)

| Feature | Definition | Positive | Rate |
|---|---|---|---|
| ***Diagnoses and other events*** | | | |
| Number of antihypertensive drug classes used for hypertension | Number of antihypertensive drug classes used for hypertension prescribed between 360 days before the index date (inclusive) and the index date (inclusive). | 7,247 | 82.8% |
| CKD stage | The stage of CKD is based on eGFR records in accordance with the Evidence-based Clinical Practice Guideline for CKD 2013 by the Japanese Society of Nephrology. | 6,854 | 78.31% |
| Heart failure diagnosis | Diagnosis of heart failure (I50, I110) between 360 days before the index date (inclusive) and the index date (inclusive). | 5,206 | 59.48% |
| History of emergency room visit | Occurrence of emergency room visit between 360 days before the index date (inclusive) and the index date (inclusive). | 2,164 | 24.73% |
| RAASi discontinuation within 1 year from 1st hyperkalemia | The patients satisfying the following conditions are assigned with 1 for this feature.<br>(a) RAASi is prescribed between 120 days before the index date (inclusive) and the index date (exclusive).<br>(b) The end date of the last RAASi administration must be on or later than the index date.<br>(c) RAASi administration is terminated at least 30 days after the index date. | 2,509 | 28.67% |
| ***Comorbidities*** | | | |
| History of myocardial infarction | Diagnosis of myocardial infarction (I21, I22, I23, I24) between 360 days before the index date (inclusive) and the index date (inclusive). | 382 | 4.36% |
| History of peripheral vascular disease | Diagnosis of peripheral vascular disease (I70, I71, I72, I73, I74, I77) between 360 days before the index date (inclusive) and the index date (inclusive). | 1,648 | 18.83% |
| History of cerebrovascular disease | Diagnosis of cerebrovascular disease (I60-I69, G45) between 360 days before the index date (inclusive) and the index date (inclusive). | 2,567 | 29.33% |
| History of dementia | Diagnosis of dementia (F00-F03, F051, G30) between 360 days before the index date (inclusive) and the index date (inclusive). | 691 | 7.9% |
| History of chronic pulmonary disease | Diagnosis of chronic pulmonary disease (J40-J47, J60-J67, J684, J701, J703, J841, J920, J961, J982, J983) between 360 days before the index date (inclusive) and the index date (inclusive). | 1,821 | 20.81% |
| History of ulcer disease | Diagnosis of ulcer disease (K221, K25-K28) between 360 days before the index date (inclusive) and the index date (inclusive). | 2,208 | 25.23% |
| History of mild liver disease | Diagnosis of mild liver disease (B18, K700-K703, K709, K71, K73, K74, K760) between 360 days before the index date (inclusive) and the index date (inclusive). | 944 | 10.79% |
| History of hemiplegia | Diagnosis of hemiplegia (G81, G82) between 360 days before the index date (inclusive) and the index date (inclusive). | 94 | 1.07% |
| History of moderate to severe liver disease | Diagnosis of moderate to severe liver disease (B150, B160, B162, B190, K704, K72, K766, I85) between 360 days before the index date (inclusive) and the index date (inclusive). | 130 | 1.49% |
| History of atrial fibrillation or atrial flutter | Diagnosis of atrial fibrillation or atrial flutter (I48) between 360 days before the index date (inclusive) and the index date (inclusive). | 1,846 | 21.09% |
| History of valvular heart disease | Diagnosis of valvular heart disease (I00-I02, I05-I09, I34, I35, I36, I37, Q20-Q25,) between 360 days before the index date (inclusive) and the index date (inclusive). | 1,347 | 15.39% |
| History of obesity | Diagnosis of obesity (E66) between 360 days before the index date (inclusive) and the index date (inclusive). | 49 | 0.56% |
| History of acute kidney injury | Diagnosis of acute kidney injury (N17) between 360 days before the index date (inclusive) and the index date (inclusive). | 385 | 4.4% |
| History of sepsis | Diagnosis of sepsis (A021, A207, A227, A241, A267, A282, A327, A394, A400-A403, A409-A415, A418-A419, A427, A548, B007, B349, B377, D71, I301, I330, J020, J209, J950, L029, L080, M8699, O080, O753, O85, O883) between 360 days before the index date (inclusive) and the index date (inclusive). | 1,161 | 13.27% |
| History of gastrointestinal bleeding | Diagnosis of gastrointestinal bleeding (K250, K252, K254, K256, K260, K262, K264, K266, K284, K290, K571, K573) between 360 days before the index date (inclusive) and the index date (inclusive). | 320 | 3.66% |
| History of peripheral oedema | Diagnosis of peripheral oedema (R600) between 360 days before the index date (inclusive) and the index date (inclusive). | 343 | 3.92% |

## (b-2) Diagnoses and medications with positive rates (2/3)

| | | | |
|---|---|---|---|
| ***Non-RAASi drugs inducing hyperkalemia*** | | | |
| Prescription of azole antifungals | Prescription of azole antifungals (J02A0) between 120 days before the index date (inclusive) and the index date (inclusive). | 49 | 0.56% |
| Prescription of calcium channel blocker | Prescription of calcium channel blocker (C08) between 120 days before the index date (inclusive) and the index date (inclusive). | 3,613 | 41.28% |
| Prescription of ciclosporin | Prescription of ciclosporin (L04X0) between 120 days before the index date (inclusive) and the index date (inclusive). | 81 | 0.93% |
| Prescription of digoxin | Prescription of digoxin (C01A1) between 120 days before the index date (inclusive) and the index date (inclusive). | 383 | 4.38% |
| Prescription of heparin | Prescription of heparin (B01B0-B01B09) between 120 days before the index date (inclusive) and the index date (inclusive). | 1,804 | 20.61% |
| Prescription of NSAIDs | Prescription of NSAIDs (M01A1, M02B0) between 120 days before the index date (inclusive) and the index date (inclusive). | 1,063 | 12.15% |
| Prescription of potassium supplements | Prescription of potassium supplements (A12B0) between 120 days before the index date (inclusive) and the index date (inclusive). | 368 | 4.2% |
| Prescription of trimethoprim | Prescription of trimethoprim (J01E0) between 120 days before the index date (inclusive) and the index date (inclusive). | 220 | 2.51% |
| Prescription of systemic corticosteroids | Prescription of systemic corticosteroids (D07B1-D07B4) between 120 days before the index date (inclusive) and the index date (inclusive). | 1,042 | 11.91% |
| ***HK treatments from the index date*** | | | |
| Treatment by thiazide diuretics | Prescription of thiazide diuretics (C03A3) between the index date (inclusive) and the outcome date (inclusive). | 906 | 10.35% |
| Treatment by loop diuretics | Prescription of loop diuretics (C03A4) between the index date (inclusive) and the outcome date (inclusive). | 4,951 | 56.57% |
| Treatment by sodium bicarbonate | Prescription of sodium bicarbonate (A02A1, K06A0, V03E0) between the index date (inclusive) and the outcome date (inclusive). | 3,271 | 37.37% |
| Treatment by potassium binder | Prescription of potassium binder (V03G1) between the index date (inclusive) and the outcome date (inclusive). | 2,556 | 29.2% |
| Treatment by glucose-insulin therapy | Prescription of glucose injection (K01B3, K01C1) and insulin (A10C0-A10C9) between the index date (inclusive) and the outcome date (inclusive). If their first prescription dates are after the index date, they must be the same. | 418 | 4.78% |

### (b-3) Diagnoses and medications with positive rates (3/3)

| Feature | Definition | Positive | Rate |
|---|---|---|---|
| ***Diabetes drugs*** | | | |
| Prescription of DPP-4i | Prescription of DPP-4i (A10N1) between 120 days before the index date (inclusive) and the index date (inclusive) | 1,516 | 17.32% |
| Prescription of SGLT-2i | Prescription of SGLT-2i (A10P1) between 120 days before the index date (inclusive) and the index date (inclusive) | 60 | 0.69% |
| Prescription of insulin | Prescription of insulin (A10C1-A10C5) between 120 days before the index date (inclusive) and the index date (inclusive) | 1,702 | 19.45% |
| Prescription of GLP-1 receptor agonist | Prescription of GLP-1 (A10S0) receptor agonist between 120 days before the index date (inclusive) and the index date (inclusive) | 79 | 0.9% |
| Prescription of fixed dose combination antidiabetics | Prescription of fixed dose combination antidiabetics (A10K2, A10K3, A10M9, A10N3, A10N9, A10P5) between 120 days before the index date (inclusive) and the index date (inclusive) | 76 | 0.87% |
| Prescription of sulfonylureas | Prescription of sulfonylureas (A10H0) between 120 days before the index date (inclusive) and the index date (inclusive) | 667 | 7.62% |
| Prescription of alpha-glucosidase inhibitor | Prescription of alpha-glucosidase inhibitor (A10L0) between 120 days before the index date (inclusive) and the index date (inclusive) | 667 | 7.62% |
| Prescription of glitazone | Prescription of glitazone (A10K1) between 120 days before the index date (inclusive) and the index date (inclusive) | 212 | 2.42% |
| Prescription of glinide | Prescription of glinide (A10M1) between 120 days before the index date (inclusive) and the index date (inclusive) | 162 | 1.85% |
| Prescription of biguanide | Prescription of biguanide (A10J1) between 120 days before the index date (inclusive) and the index date (inclusive) | 529 | 6.04% |
| ***Dyslipidemia drugs*** | | | |
| Prescription of statin | Prescription of statin between 120 days before the index date (inclusive) and the index date (inclusive) | 2,596 | 29.66% |
| Prescription of fibrate | Prescription of fibrate between 120 days before the index date (inclusive) and the index date (inclusive) | 150 | 1.71% |
| Prescription of bile acid sequestrant | Prescription of bile acid sequestrant between 120 days before the index date (inclusive) and the index date (inclusive) | 9 | 0.1% |
| Prescription of cholesterol absorption inhibitor | Prescription of cholesterol absorption inhibitor between 120 days before the index date (inclusive) and the index date (inclusive) | 387 | 4.42% |
| Prescription of other antilipidemic drugs | Prescription of other antilipidemic drugs between 120 days before the index date (inclusive) and the index date (inclusive) | 322 | 3.68% |
| ***Treatment for hypertension*** | | | |
| Prescription of ARB or ACEi | Prescription of ARB or ACEi between 120 days before the index date (inclusive) and the index date (inclusive) for hypertension patients | 4,457 | 50.93% |
| ***Treatments for heart failure*** | | | |
| Prescription of beta blocker | Prescription of beta blocker (C07) between 120 days before the index date (inclusive) and the index date (inclusive) for heart failure patients | 2,375 | 27.14% |
| Prescription of inotropes | Prescription of inotropes (C01F0) between 120 days before the index date (inclusive) and the index date (inclusive) for heart failure patients | 949 | 10.84% |
| Prescription of MRA | Prescription of MRA (C03A1) between 120 days before the index date (inclusive) and the index date (inclusive) for heart failure patients | 1,820 | 20.8% |

**Table S2. Definitions of primary and secondary clinical outcomes**

| Clinical outcomes | Definition in the model derivation and internal validation sets | Definition in the external validation set | Positive rate (%) in derivation set (N=8,752) | Positive rate (%) in internal validation set (N=4,990) | Positive rate (%) in external validation set (N=86,279) |
|---|---|---|---|---|---|
| All-cause death | Death within 1,080 days after the first hyperkalemic episode | Same as derivation and internal validation sets | 16.59 | 16.83 | 11.92 |
| Introduction of renal replacement therapy | Any procedures related to kidney transplant or dialysis within 1,080 days after the first episode of elevated serum potassium | Same as derivation and internal validation sets | 10.13 | 14.83 | 5.70 |
| Hospitalization for heart failure | Hospitalizations with heart failure diagnosis as a main reason for the hospitalization within 1,080 days after the first hyperkalemic episode | Hospitalizations with a heart failure diagnosis within first 7 days regardless of main reason for the hospitalization | 15.37 | 14.81 | 10.51 |
| Cardiovascular events | Hospitalization with cardiovascular event (a composite of myocardial infarction, arrhythmia, cardiac arrest, and stroke) diagnosis as a main reason for the hospitalization within 1,080 days after the first hyperkalemic episode | Hospitalizations with a cardiovascular event diagnosis within first 7 days regardless of the main reason for the hospitalization | 7.10 | 8.84 | 10.32 |

**Table S3. Definitions of exploratory clinical outcomes**

| Clinical outcomes | Definition in the model derivation and internal validation sets |
|---|---|
| All cause hospitalization | The first hospitalization within 1,080 days after the first hyperkalemia episode |
| Introduction of dialysis | The first dialysis within 1,080 days after the first hyperkalemic episode |
| Emergency room visit | The first occurrence of emergency room visits within 1,080 days after the first hyperkalemic episode |
| Hospitalization with intensive care unit admission | The first occurrence of admission to intensive care unit within 1,080 days after the first hyperkalemic episode |

**Table S4. Tuning hyperparameters for each modeling algorithm**

**(a) Hyperparameters for the extreme gradient boosting model**

| Name | Description | Search spaces | | Best params | | | |
|---|---|---|---|---|---|---|---|
| | | Distribution | Range | All-cause death | RRT | Hospitalization for heart failure | Cardiovascular event |
| colsample_bytree | Subsample ratio of columns when constructing each tree | Uniform | 0.1:0.9 | 0.57 | 0.32 | 0.35 | 0.59 |
| early_stop | Early stopping to avoid overfitting | Categorical | True, False | FALSE | FALSE | FALSE | FALSE |
| gamma | Minimum loss reduction required to make a node split | Log-uniform | 0:0.5 | 1.43 | 1.90 | 2.20 | 3.02 |
| learning rate | Learning rate | Log-uniform | -3:0 | 0.003 | 0.003 | 0.033 | 0.010 |
| max_depth | Maximum depth of each tree | Discrete uniform | 3:14 | 9 | 4 | 10 | 11 |
| min_child_weight | Minimum sum of instance weight (hessian) needed in a child | Discrete uniform | 0.1:0.9 | 4 | 6 | 6 | 8 |
| n_estimators | Number of boosted trees to fit | Discrete uniform | 100:9,999 | 6,706 | 4,471 | 2,847 | 7,904 |
| reg_alpha | L1 regularization term on weights | Log-uniform | -5:2 | 9.20 | 18.05 | 0.04 | 10.62 |
| reg_lambda | L2 regularization term on weights | Log-uniform | -3:2 | 20.44 | 0.01 | 96.63 | 0.02 |
| scale_pos_weight | Balancing of positive and negative weights | - | 1, neg/pos rate, square-root of neg/pos rate | 1.00 | 8.87 | 1.00 | 1.00 |
| tree_method | Tree construction algorithm | Categorical | auto, hist | auto | hist | auto | auto |

neg: negative, pos: positive,

auto: use heuristic to choose the fastest method, hist: faster histogram optimized approximate greedy algorithm.

**(b) Hyperparameters for the logistic regression model**

| Name | Description | Search spaces | | Best parameters | | | |
|---|---|---|---|---|---|---|---|
| | | Distribution | Range | All-cause death | RRT | Hospitalization for heart failure | Cardiovascular event |
| C | Inverse of regularization strength | Log-uniform | -3:1 | 1.98 | 0.05 | 0.89 | 0.45 |
| penalty | Regularization | Categorical | L1, L2 | L1 | L1 | L1 | L1 |

**(c) Hyperparameters for the neural network model**

| Name | Description | Search spaces | | Best parameters | | | |
|---|---|---|---|---|---|---|---|
| | | Distribution | Range | All-cause death | RRT | Hospitalization for heart failure | Cardiovascular event |
| epochs | Number of times each patient used during training | Discrete uniform | 1:30 | 29 | 18 | 6 | 7 |
| batch_size | Number of patients used for each parameter update | Discrete uniform | 32, 64 | 64 | 32 | 32 | 32 |

**Table S5. Prediction performance of the machine learning models for exploratory outcomes on the internal validation set**

| Outcome | ML algorithm | AUROC | Sensitivity | Specificity | PPV | NPV |
|---|---|---|---|---|---|---|
| **Cut-off = 0.5** | | | | | | |
| All cause hospitalization | XGB | 0.861 | 0.788 | 0.773 | 0.923 | 0.511 |
| | LR | 0.840 | 0.941 | 0.404 | 0.846 | 0.664 |
| | NN | 0.800 | 0.895 | 0.465 | 0.854 | 0.559 |
| Introduction of dialysis | XGB | 0.957 | 0.828 | 0.942 | 0.712 | 0.969 |
| | LR | 0.947 | 0.615 | 0.967 | 0.762 | 0.935 |
| | NN | 0.950 | 0.732 | 0.957 | 0.750 | 0.954 |
| Emergency room visit | XGB | 0.761 | 0.516 | 0.836 | 0.668 | 0.729 |
| | LR | 0.746 | 0.489 | 0.838 | 0.659 | 0.719 |
| | NN | 0.689 | 0.550 | 0.723 | 0.561 | 0.715 |
| Hospitalization with ICU admission | XGB | 0.802 | 0.671 | 0.787 | 0.264 | 0.955 |
| | LR | 0.782 | 0.110 | 0.993 | 0.644 | 0.907 |
| | NN | 0.764 | 0.624 | 0.765 | 0.233 | 0.947 |

ML, machine learning; AUROC, area under the receiver operator characteristic curve; PPV, positive predictive value; NPV, negative predictive value; XGB, extreme gradient boosting; LR, logistic regression; NN, neural network; ICU, intensive care unit.

**Table S6. Prediction performance of the extreme gradient boosting models on the external validation set based on the restricted condition***

| Outcome | AUROC | Sensitivity | Specificity | PPV | NPV |
|---|---|---|---|---|---|
| All-cause death | 0.711 | 0.663 | 0.647 | 0.150 | 0.953 |
| Introduction of RRT | 0.867 | 0.427 | 0.928 | 0.210 | 0.973 |
| Hospitalization for HF | 0.662 | 0.461 | 0.751 | 0.162 | 0.930 |
| Cardiovascular events | 0.586 | 0.417 | 0.697 | 0.117 | 0.926 |

*Analysis restricting the data collection period for input variables within one month after the first hyperkalemic episode and predicting the risk of clinical outcomes after the data collection period.

ML, machine learning; AUROC, area under the receiver operator characteristic curve; PPV, positive predictive value; NPV, negative predictive value; HF, heart failure; RRT, renal replacement therapy.

Calibration analysis was made based on the best-cut off values. The best cut-off point was set as the cut-off value to the point on the ROC curve that maximizes the sum of sensitivity + specificity – 1 i.e. the Youden index that provides efficient tradeoff between sensitivity and specificity.

## Table S7. TRIPOD Checklist

| Section/Topic | Item | D/V* | Checklist Item | Section/Paragraph number |
|---|---|---|---|---|
| Title | 1 | D;V | Identify the study as developing and/or validating a multivariable prediction model, the target population, and the outcome to be predicted. | Title page |
| Abstract | 2 | D;V | Provide a summary of objectives, study design, setting, participants, sample size, predictors, outcome, statistical analysis, results, and conclusions. | Abstract |
| Background and objectives | 3a | D;V | Explain the medical context (including whether diagnostic or prognostic) and rationale for developing or validating the multivariable prediction model, including references to existing models. | Introduction / #2 |
| | 3b | D;V | Specify the objectives, including whether the study describes the development or validation of the model or both. | Introduction / #4 |
| Source of data | 4a | D;V | Describe the study design or source of data (e.g., randomized trial, cohort, or registry data), separately for the development and validation data sets, if applicable. | Methods, Study design, patient selection, and data handling / #1 |
| | 4b | D;V | Specify the key study dates, including start of accrual; end of accrual; and, if applicable, end of follow-up. | Methods, Study design, patient selection, and data handling / #1, #3 |
| Participants | 5a | D;V | Specify key elements of the study setting (e.g., primary care, secondary care, general population) including number and location of centers. | Supplementary, Method details, Data sources |
| | 5b | D;V | Describe eligibility criteria for participants. | Methods, Study design, patient selection, and data handling / #2 |
| | 5c | D;V | Give details of treatments received, if relevant. | Methods, Risk factors and outcomes / #1 |
| Outcome | 6a | D;V | Clearly define the outcome that is predicted by the prediction model, including how and when assessed. | Supplementary, Table S2 |
| | 6b | D;V | Report any actions to blind assessment of the outcome to be predicted. | N/A because the data source is existing database |
| Predictors | 7a | D;V | Clearly define all predictors used in developing or validating the multivariable prediction model, including how and when they were measured. | Supplementary, Table S1 |
| | 7b | D;V | Report any actions to blind assessment of predictors for the outcome and other predictors. | N/A because the data source is existing database |
| Sample size | 8 | D;V | Explain how the study size was arrived at. | Methods, Study design, patient selection, and data handling / #1, #3 |
| Missing data | 9 | D;V | Describe how missing data were handled (e.g., complete-case analysis, single imputation, multiple imputation) with details of any imputation method. | Supplementary, Method details, Machine learning algorithms |
| Statistical analysis methods | 10a | D | Describe how predictors were handled in the analyses. | Supplementary, Method details, Machine learning algorithms |
| | 10b | D | Specify type of model, all model-building procedures (including any predictor selection), and method for internal validation. | Methods, Machine learning algorithms / #1, #2, Supplementary, Method details, Machine learning algorithms |
| | 10c | V | For validation, describe how the predictions were calculated. | Methods, Validation / #2 |
| | 10d | D;V | Specify all measures used to assess model performance and, if relevant, to compare multiple models. | Methods, Validation / #1 |

| | | | | |
|---|---|---|---|---|
| | 10e | V | Describe any model updating (e.g., recalibration) arising from the validation, if done. | N/A because no updating was done |
| Risk groups | 11 | D;V | Provide details on how risk groups were created, if done. | N/A because no risk group was created |
| Development vs. validation | 12 | V | For validation, identify any differences from the development data in setting, eligibility criteria, outcome, and predictors. | Methods, Validation / #2 |
| Participants | 13a | D;V | Describe the flow of participants through the study, including the number of participants with and without the outcome and, if applicable, a summary of the follow-up time. A diagram may be helpful. | Figure 1, Table 1, Supplementary, table S1 |
| | 13b | D;V | Describe the characteristics of the participants (basic demographics, clinical features, available predictors), including the number of participants with missing data for predictors and outcome. | Table 1, Supplementary, table S1 |
| | 13c | V | For validation, show a comparison with the development data of the distribution of important variables (demographics, predictors and outcome). | Table 1 |
| Model development | 14a | D | Specify the number of participants and outcome events in each analysis. | Supplementary, table S2 |
| | 14b | D | If done, report the unadjusted association between each candidate predictor and outcome. | N/A because no unadjusted association was calculated |
| Model specification | 15a | D | Present the full prediction model to allow predictions for individuals (i.e., all regression coefficients, and model intercept or baseline survival at a given time point). | N/A because the main model was developed by XGBoost then cannot represented by set of numeric coefficients |
| | 15b | D | Explain how to use the prediction model. | N/A because no public tool of calculator was developed yet. |
| Model performance | 16 | D;V | Report performance measures (with CIs) for the prediction model. | Results, Model derivation and internal validation / #1, External validation / #1 |
| Model-updating | 17 | V | If done, report the results from any model updating (i.e., model specification, model performance). | N/A because no updating was done |
| Limitations | 18 | D;V | Discuss any limitations of the study (e.g., nonrepresentative sample, few events per predictor, missing data). | Discussion, Strengths, and limitations / #1, #2, #3 |
| Interpretation | 19a | V | For validation, discuss the results with reference to performance in the development data, and any other validation data. | Discussion / #2 |
| | 19b | D;V | Give an overall interpretation of the results, considering objectives, limitations, results from similar studies, and other relevant evidence. | Discussion / #1, #2 |
| Implications | 20 | D;V | Discuss the potential clinical use of the model and implications for future research. | Discussion / #4 |
| Supplementary information | 21 | D;V | Provide information about the availability of supplementary resources, such as study protocol, Web calculator, and data sets. | N/A because study protocol nor data sets are publicly available |
| Funding | 22 | D;V | Give the source of funding and the role of the funders for the present study. | Abstract, Funding |

*Items relevant only to the development of a prediction model are denoted by D, items relating solely to the validation of a prediction model are denoted by V, and items relating to both are denoted D;V.

**Figure S1. Data handling of the internal dataset.**

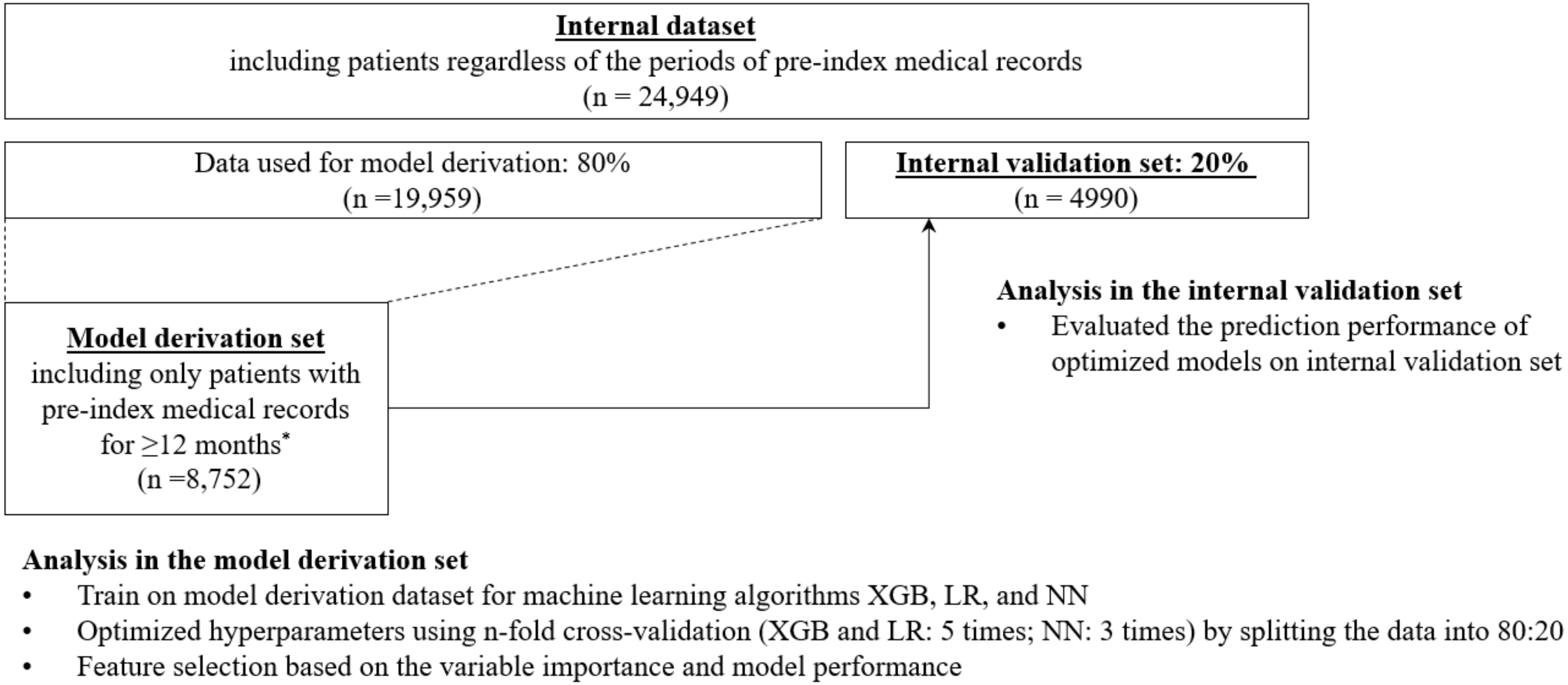


XGB, extreme gradient boosting; LR, logistic regression; NN, neural network.
*The model derivation set included only patients with at least 12-month pre-index medical records to ensure the rigorous evaluation of the patient background and medical history.

**Figure S2. Patient flow diagrams for external validation.**

**(a) Original condition** **(b) Restricted condition**

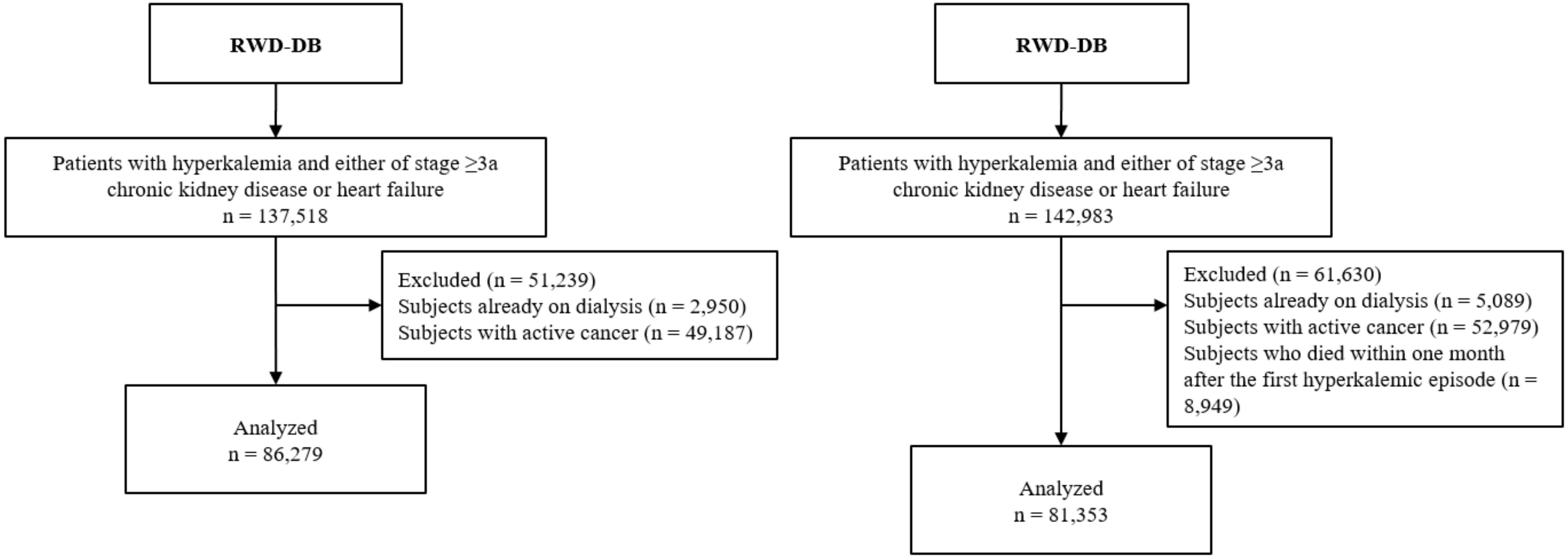

**Figure S3. Receiver operator characteristics curve for exploratory outcomes evaluated on the internal dataset.**

XGB, extreme gradient boosting; LR, logistic regression; NN, neural network; AUC, area under the operator receiver characteristics curve; ICU, intensive care unit.

**(a) All-cause hospitalization**

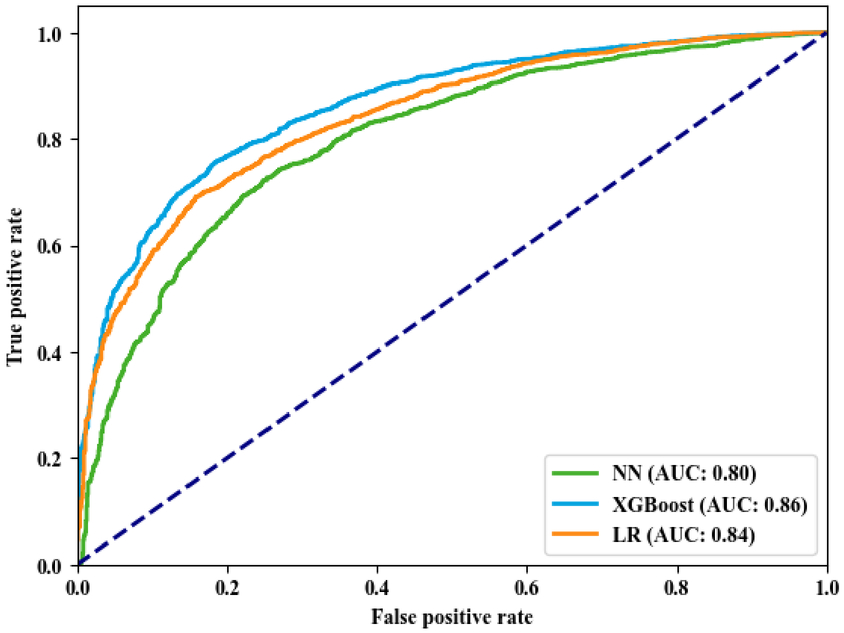


**(b) Introduction of dialysis**

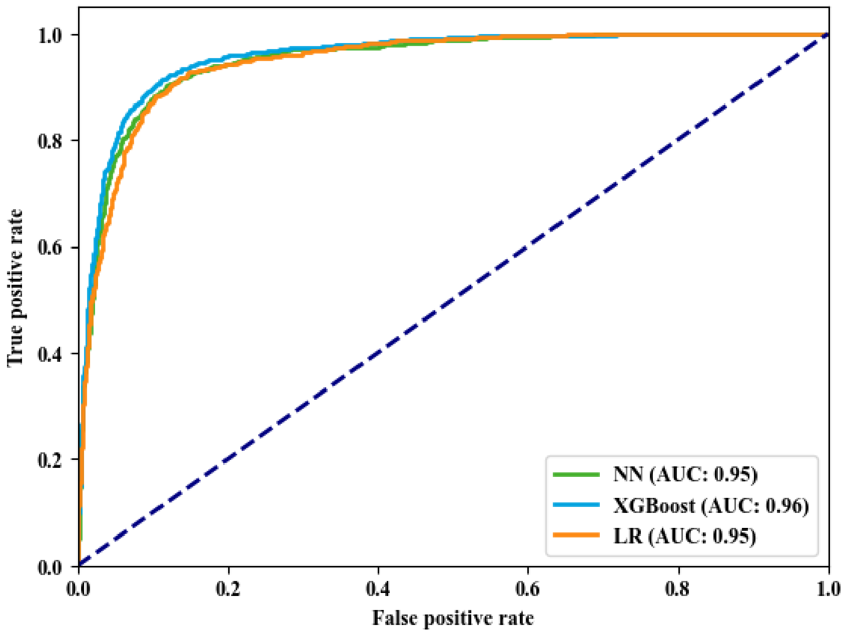

**(c) Emergency room visit**

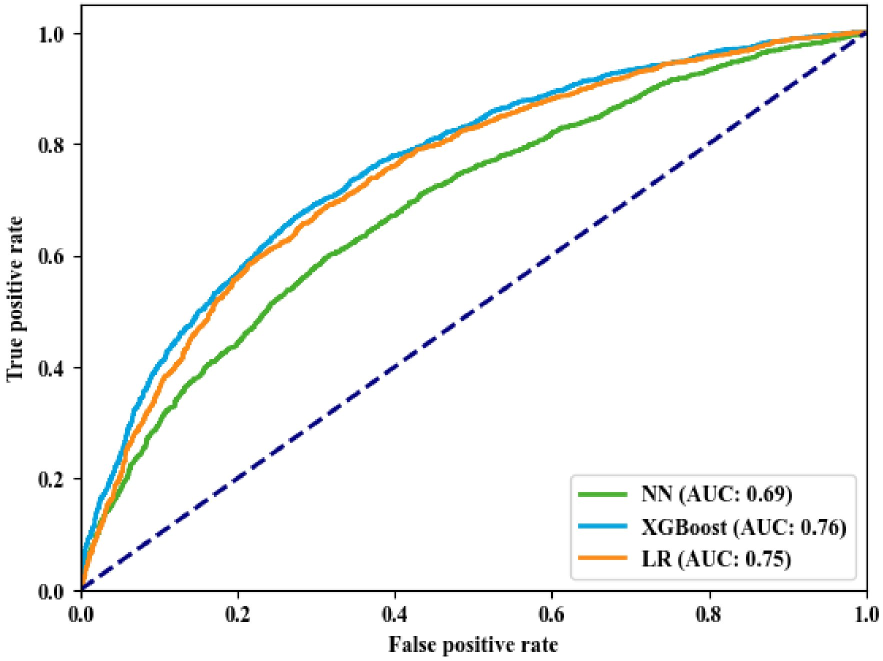


**(d) Hospitalization with ICU admission**

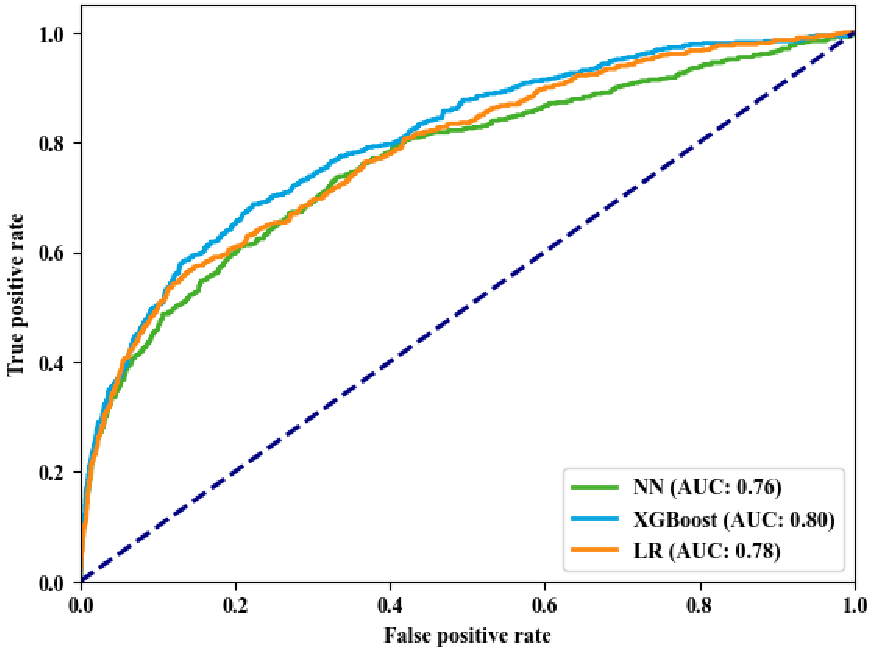

**Figure S4 Receiver operator characteristic curves of the machine learning model in the external validation set.**

**(a) Original condition**

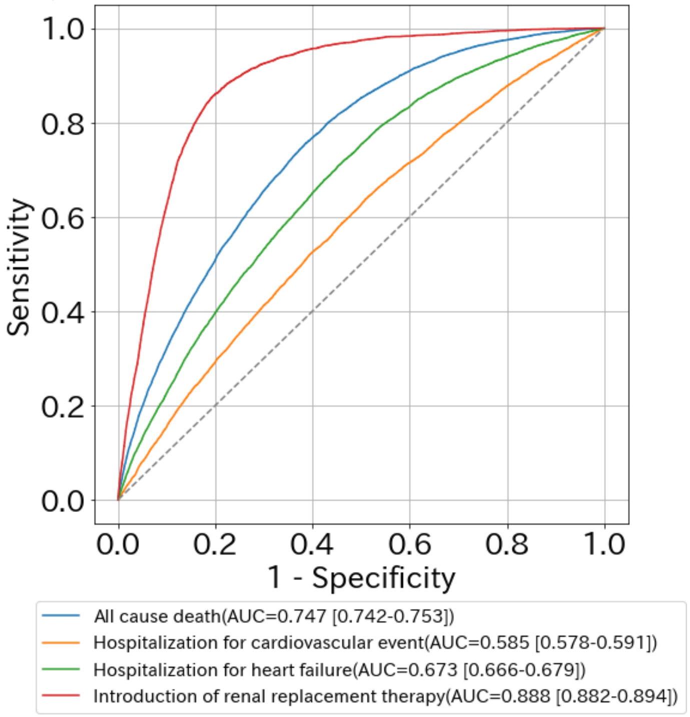


**(b) Restricted condition**

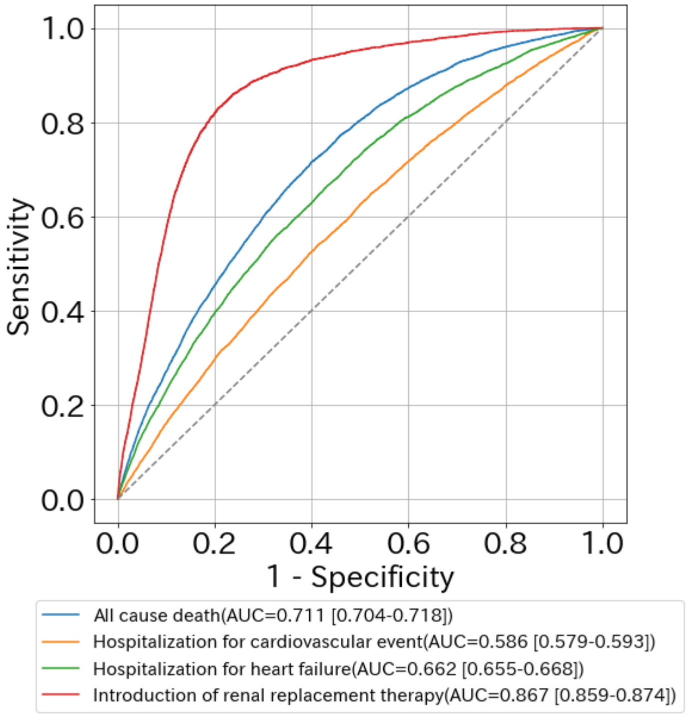

**Figure S5. Sensitivity analysis in the subgroup of patients in the external validation set applying the same outcome definition used in the derivation set.**

**(a) Patient flow diagram**

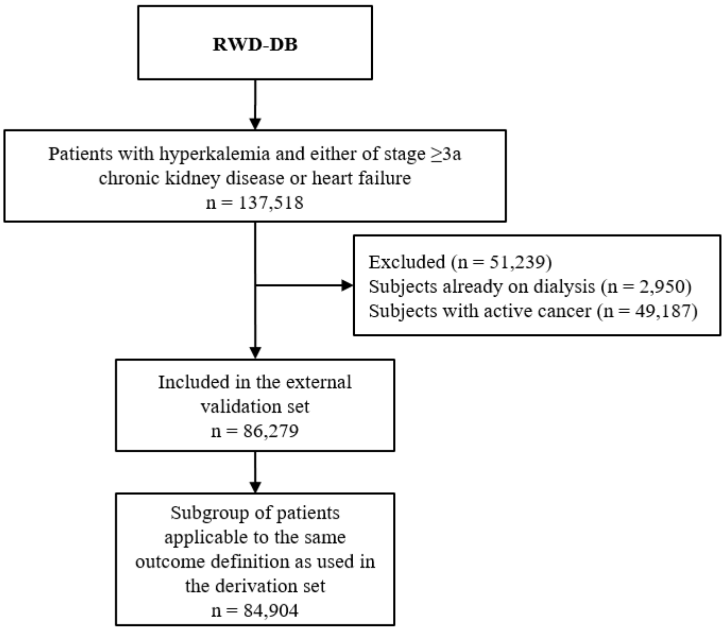


**(b) Receiver operator characteristics curve**

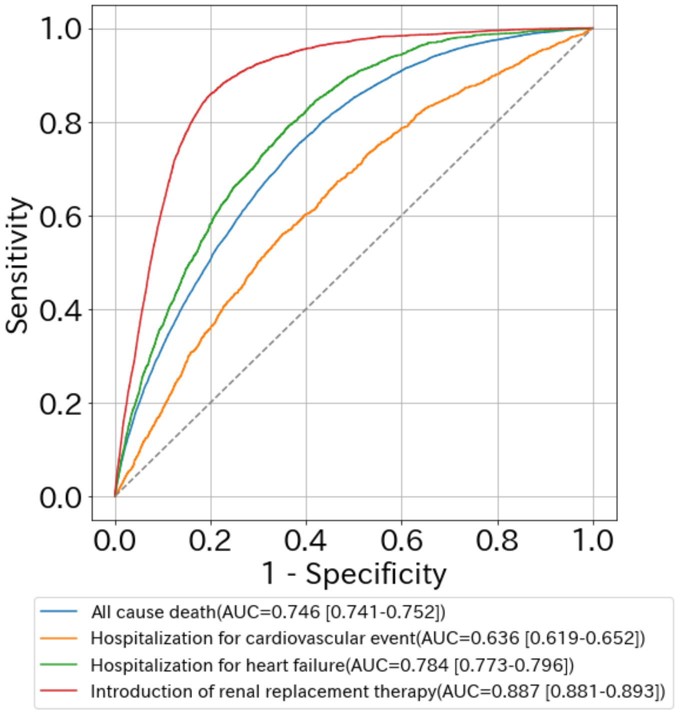

**(c) Kaplan-Meier plots of high- and low-risk groups based on risk predictions**

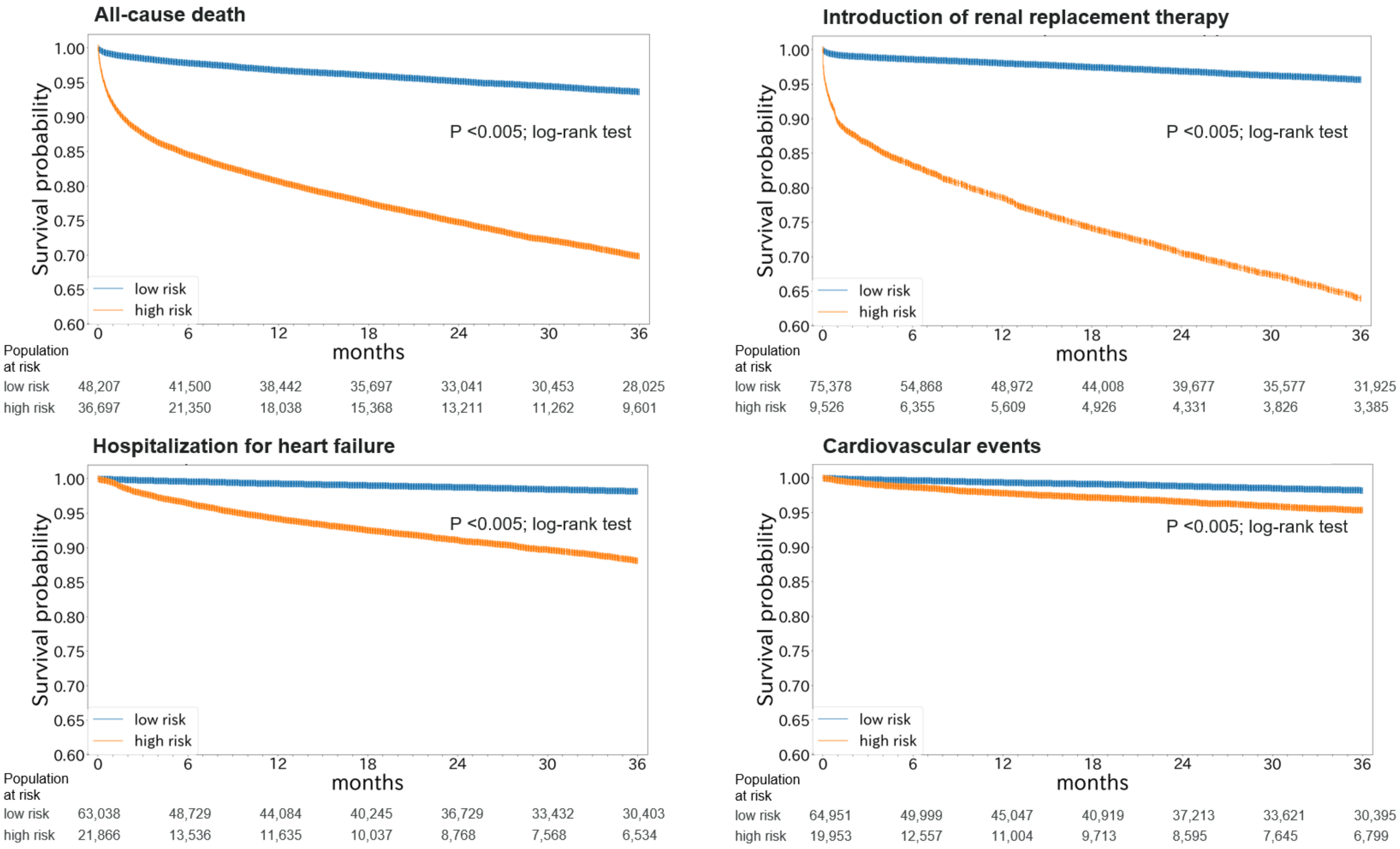

**Fig. S6. Kaplan-Meier plots of high- and low-risk groups based on risk predictions in the external validation set based on the restricted condition*.**

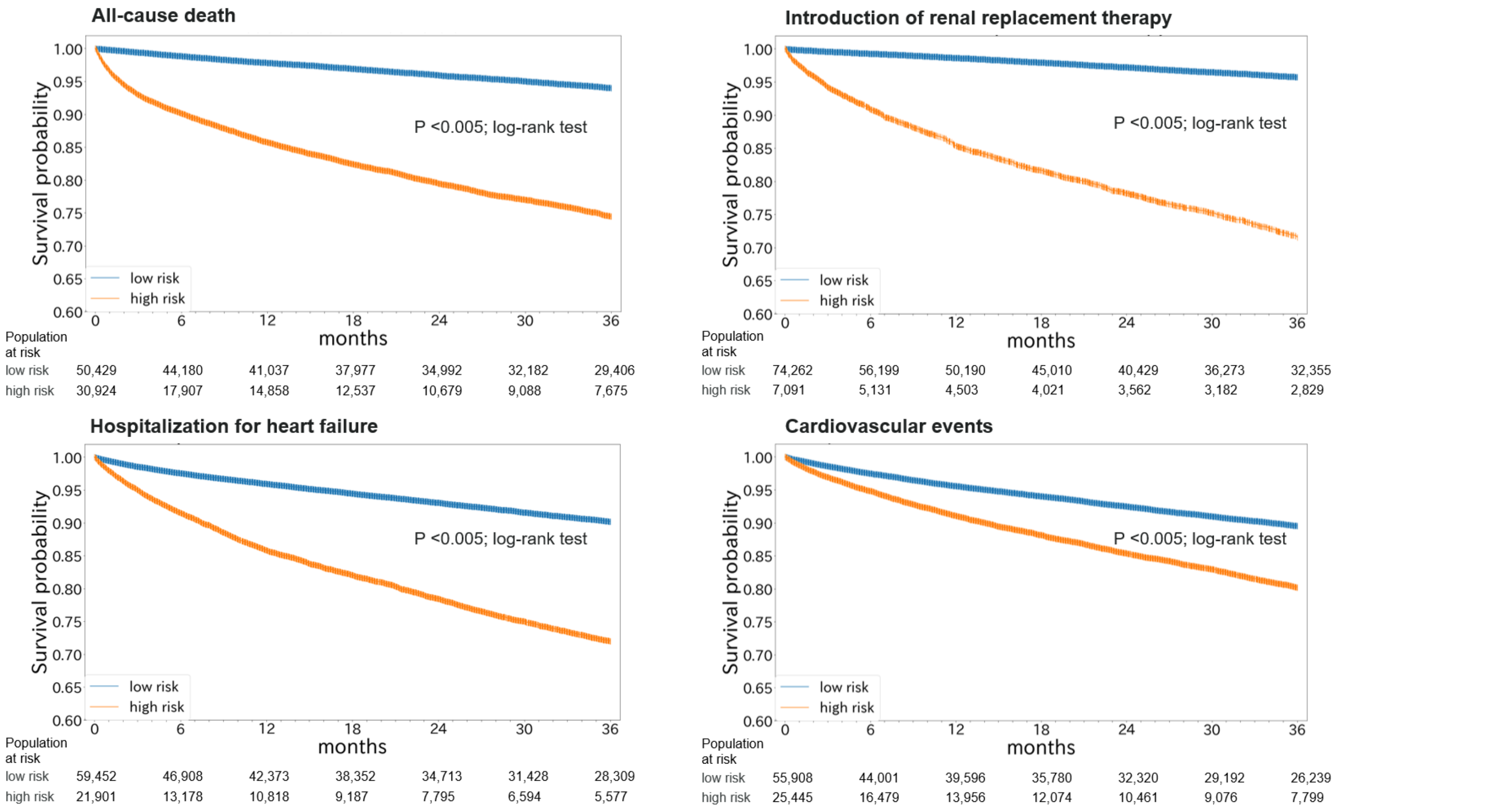


*Analysis restricting the data collection period for input variables within one month after the first hyperkalemic episode and predicting the risk of clinical outcomes after the data collection period.

## References


1. Sagi O, Rokach L. Ensemble learning: A survey. *Wiley Interdisciplinary Reviews: Data Mining and Knowledge Discovery* 2018; **8**(4): e1249.
2. Agarap AF. Deep learning using rectified linear units (relu). *arXiv preprint arXiv:180308375* 2018.
3. Lundberg, SM and Lee, SI. A unified approach to interpreting model predictions. In Proceedings of the 31st international conference on neural information processing systems 2017: pp. 4768-4777.
4. SHAP. https://github.com/slundberg/shap. (accessed Aug 2021)
5. Lundberg, SM, Erion, G, Chen H, et al. From local explanations to global understanding with explainable AI for trees. Nature machine intelligence, 2020; 2(1): 56-67.
6. Collins GS, Reitsma JB, Altman DG, and Moons KG. Transparent reporting of a multivariable prediction model for individual prognosis or diagnosis (TRIPOD): the TRIPOD statement. *BMJ (Clinical research ed)* 2015; **350**: g7594.
